# A client–server framework for 3D remote visualization of radiotherapy treatment space

**DOI:** 10.3389/fonc.2013.00018

**Published:** 2013-02-22

**Authors:** Anand P. Santhanam, Yugang Min, Tai H. Dou, Patrick Kupelian, Daniel A. Low

**Affiliations:** Department of Radiation Oncology, University of CaliforniaLos Angeles, CA, USA

**Keywords:** remote visualization, radiotherapy, 3D monitoring, patient positioning, client–server architecture

## Abstract

Radiotherapy is safely employed for treating wide variety of cancers. The radiotherapy workflow includes a precise positioning of the patient in the intended treatment position. While trained radiation therapists conduct patient positioning, consultation is occasionally required from other experts, including the radiation oncologist, dosimetrist, or medical physicist. In many circumstances, including rural clinics and developing countries, this expertise is not immediately available, so the patient positioning concerns of the treating therapists may not get addressed. In this paper, we present a framework to enable remotely located experts to virtually collaborate and be present inside the 3D treatment room when necessary. A multi-3D camera framework was used for acquiring the 3D treatment space. A client–server framework enabled the acquired 3D treatment room to be visualized in real-time. The computational tasks that would normally occur on the client side were offloaded to the server side to enable hardware flexibility on the client side. On the server side, a client specific real-time stereo rendering of the 3D treatment room was employed using a scalable multi graphics processing units (GPU) system. The rendered 3D images were then encoded using a GPU-based H.264 encoding for streaming. Results showed that for a stereo image size of 1280 × 960 pixels, experts with high-speed gigabit Ethernet connectivity were able to visualize the treatment space at approximately 81 frames per second. For experts remotely located and using a 100 Mbps network, the treatment space visualization occurred at 8–40 frames per second depending upon the network bandwidth. This work demonstrated the feasibility of remote real-time stereoscopic patient setup visualization, enabling expansion of high quality radiation therapy into challenging environments.

## INTRODUCTION

Radiotherapy is safely employed for treating wide variety of cancers. The radiotherapy workflow includes the positioning of the patient in the intended treatment position. Trained radiation therapists conduct this, but occasionally consultation is required from other experts, including the radiation oncologist, dosimetrist, or medical physicist. In many circumstances, including rural clinics and developing countries, this expertise is not immediately available, so the concerns of the treating therapists may not get addressed. By the year 2015, 15 million new cancer patients are expected in the world each year, of which 10 million will be in the developing countries. Ensuring that those patients receive appropriate treatment is a major challenge (Bhadrasain, 2005; National Cancer Institute, 2010; International Agency for Research on Cancer, 2012). Between 2005 and 2025, 100 million cancer victims in the developing countries will require radiotherapy, for cure or the relief of symptoms such as pain and bleeding. However, the lack of radiotherapy treatment expertise in those countries leads to only 20–25% of patients in developing countries being treated with radiotherapy (Bhadrasain, 2005). This situation will only worsen in the future unless steps are taken to address it.

Radiation therapy treatments continue to gain complexity and modern linear accelerators are essentially robotically controlled, creating the need for more advanced in-room monitoring. Current monitoring is restricted to one or more 2D video cameras positioned in the room and monitored by the radiation therapists. There are neither computer-based analysis nor monitoring of the video, they are intended as straightforward monitoring devices because the therapists cannot be in the room during treatment and the radiation shielding requirements preclude the use of windows.

One of the challenges of modern radiation therapy is the distribution of specific expertise required for each clinic to safely treat their patients. Medical physicists, for example, are often called to the treatment room to assist the radiation therapists in evaluating a treatment set up. In many clinics, especially rural clinics, there are not enough medical physicists to allow full-time access, and the therapists will not have access to the expertise. This problem also exists in treatment planning expertise. Recent advances in the digital storage and efficient and reliable communications have enabled improved 2D remote visualization that facilitated a decentralized radiotherapy services by allowing remote quality assurance of treatment delivery (Olsen et al., 2000). An early work toward such a 3D collaborative radiotherapy workflow was developed at the departments of radiotherapy at the University Hospital of North Norway and the Norwegian Radium Hospital (Norum et al., 2005). The treatment planning systems at the two institutions were connected through a 2 Mbps digital telecommunication line and 2D videoconferencing units were installed. The feasibility of performing clinical operations such as treatment planning, supervision, second opinions, and education using the collaborative system were investigated for two dummy cases and six patients. Remote treatment simulation procedures were carried out for five patients and a cost-minimization analysis was performed. It was observed that 2D remote supervision was possible with the threshold (break-even point) comparing the costs of such visualization to be 12 patients/year.

Virtual reality-based visualizations greatly help in developing 3D collaborative environments for radiotherapy applications. Efforts by peers have focused on developing visualization frameworks specifically for radiotherapy training (Phillips et al., 2008), planning (Geng, 2008), and treatment simulation (Santhanam et al., 2008). While 3D visualization has assisted in the planning and simulation process, it has not been used in the treatment room.

Our context in this paper is focused on real-time acquisition and visualization of the patient treatment setup for radiotherapy. While expertise that is required in the treatment room can be given over the telephone, it will be much less effective than having the expert physically within the room. We hypothesize that 3D visualization of the patient setup and intrafraction motion will enable experts to provide important consultative services to rural and developing country clinics. In this paper, we present an approach for remote visualizing in 3D the patient setup, allowing the expert to interact with the local team as though they were in the linear accelerator room with them. The key contribution of this paper is to present a real-time remote multi-3D camera-based imaging system that provides remote real-time 3D images of the patient positioning setup at greater than 30 frames per second (FPS) required for effective visualization (Hamza-lup et al., 2007).

## MATERIALS AND METHODS

### MULTI-3D CAMERA SETUP

**FIGURE 1:**
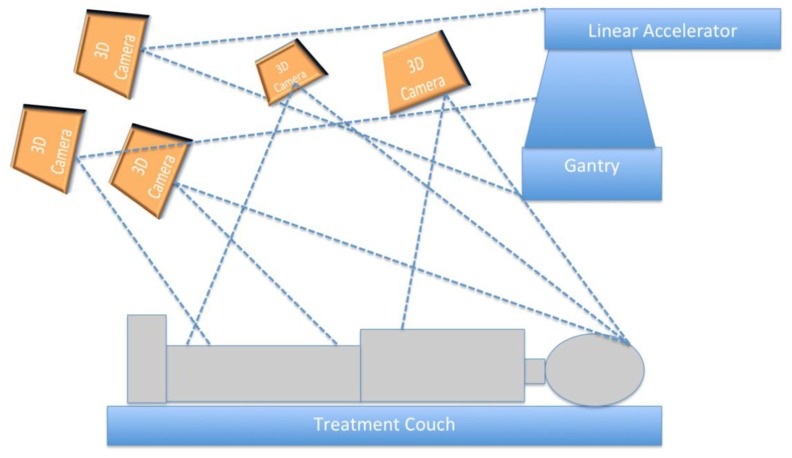
**A schematic representation of the camera setup for head and neck radiotherapy**.

**Figure 1** presents the schematic representation of the proposed 3D patient and treatment setup monitoring system. As in a typical external beam radiotherapy setup, a patient is illustrated to lie on a treatment couch with the linear accelerator gantry targeting the region of interest. A set of 3D cameras were used for illustration purposes to acquire the treatment environment. For our setup, the cameras were distributed throughout the room to capture the entire patient, couch, and gantry and to minimize gaps in the images caused by occlusions. The cameras were connected to a controller program running on a computer through universal serial bus (USB) ports.

For this work, we selected the Kinect camera as the 3D camera used for the proposed 3D monitoring system as it was cost effective and readily available across the world. From a technical perspective, Kinect cameras provide both color and depth information, which enables to use the wide range of vision and 3D information processing algorithms for monitoring and visualization purposes. These cameras were being sold as part of a computer gaming system, so they were not required to be quantitative. However, in this application, the images from multiple cameras needed to be stitched together to form a seamless and visually accurate representation of the room environment. If the cameras were not accurately calibrated, the surfaces from two cameras would not coincide where their image fields overlapped. This led to the development of a calibration procedure (Santhanam et al., in review).

The camera calibration was performed for each camera to determine the relationship between the raw depth information and the calibrated depth. Images from each camera were first corrected for camera-specific distortion. Image refining steps prepared the 3D information (2D color and depth information) from each of the cameras into a single 3D context. Image stitching was performed by computing the transformation matrix that transforms the 3D information of each camera into a specific reference camera. The 3D stitched images represented the patient setup on the treatment couch along with the gantry and couch positions.

### CLIENT–SERVER ARCHITECTURE

**FIGURE 2:**
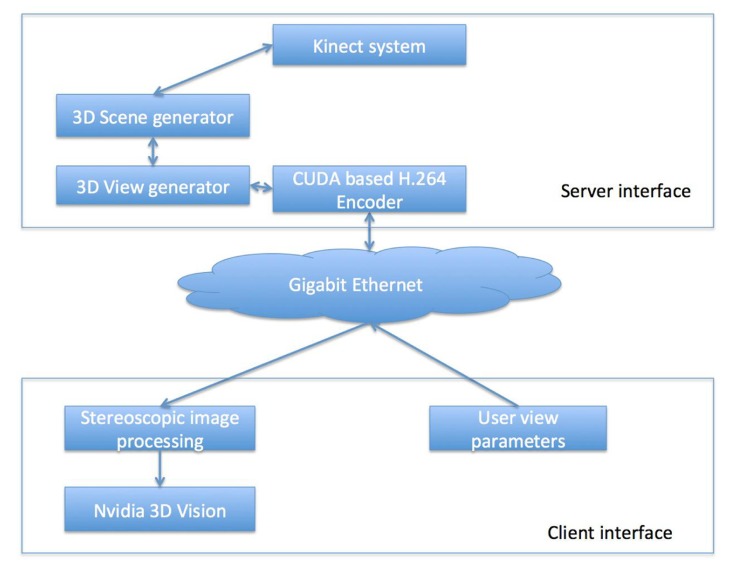
**A schematic representation of the proposed tracking system for head and neck radiotherapy**.

A client and server system was used to correct, stitch, and transport the images in real-time to the observer. The term client refers to the user interface associated with each remotely located expert. The term server relates to the software interface that controls the multi 3D Kinect camera system discussed in the “Multi-3D Camera Setup” section. **Figure 2** schematically represents the client–server setup used for the 3D visualization. Such a setup enables multiple remotely located experts to simultaneously visualize the 3D content. The client and the server interface are now described.

#### Client interface

For our proposed visualization system, we offloaded the rendering task to the server to allow the 3D content to be visualized at 30 FPS for satisfying real-time requirements (Hamza-lup et al., 2007). In order to achieve this for every client, each client was associated with a session ID that uniquely identified the client and the associated stereoscopic visualization parameters, which were the eye location and orientation in the treatment room global coordinates, the gaze direction associated with each eye, the 3D clipping boundary, 3D zoom factor and a user-provided value that selected the image compression quality. Each session ID was also associated with a desired visualization frame rate, which, coupled with the network traffic rate, determined the 3D resolution of the visualized space. A TCP-based connection (Stevens, 2003) was used between the client and the server. Once the connection was established, the client interface sent a “heartbeat” message to the server providing the visualization parameters. The server generated the stereoscopic image and sends it back to the client.

#### Server interface

**FIGURE 3:**
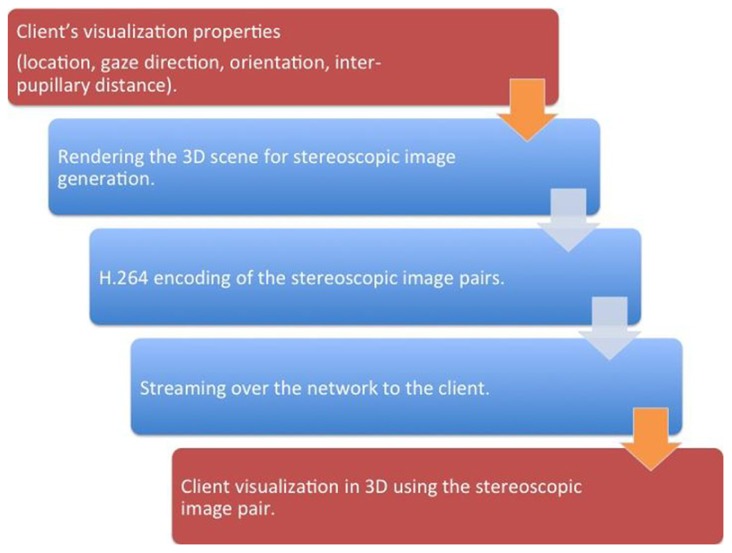
**A schematic representation of the client–server interfaces**. The steps that occur on the client is represented by red while the steps that occur on the server side is represented by blue. The brown arrows represent the client–server network interface.

The server interface consisted of three pipelined procedural threads. The first procedural thread was used to creating a connection between the client and the server. The thread also maintained the frame rate desired by the client. The second procedural thread acquired the 3D treatment room space as discussed in “Multi-3D Camera Setup” section and the third procedural thread communicated with the client for sending the 2D stereoscopic projections of the treatment room space. **Figure 3** presents a schematic representation of the client–server interface. For each client identified by its session ID, the stereoscopic visualization properties were retrieved and, using a GPU-based render-to-texture feature, the 3D scene was projected for each eye and its clipping properties to form a 2D image (Shreiner, 2009).

The steps involved in the render-to-texture feature are as follows. First the 3D treatment room space, in the form of a vertex list, was transferred to one of the GPU’s memory. We then assigned a texture memory space as the location where the final rendered image would be placed. We then created an openGL pipeline that processed the 3D vertex list for each eye and clipping boundary specifications provided by the client. Finally, using the GPU’s vector functionality, we projected each 3D location in the vertex list through the OpenGL pipeline onto the pre-determined 2D texture. The 2D texture was then copied from the GPU into the server (Shreiner, 2009).

The 2D images were losslessly encoded in the H.264 stream format, which reduced the network-transmitted 2D image data size. The encoded 2D images for each eye were then transmitted to the client interface, which were subsequently decoded and visualized using Nvidia’s 3D vision interface.

#### GPU-based H.264 video encoding

**FIGURE 4:**
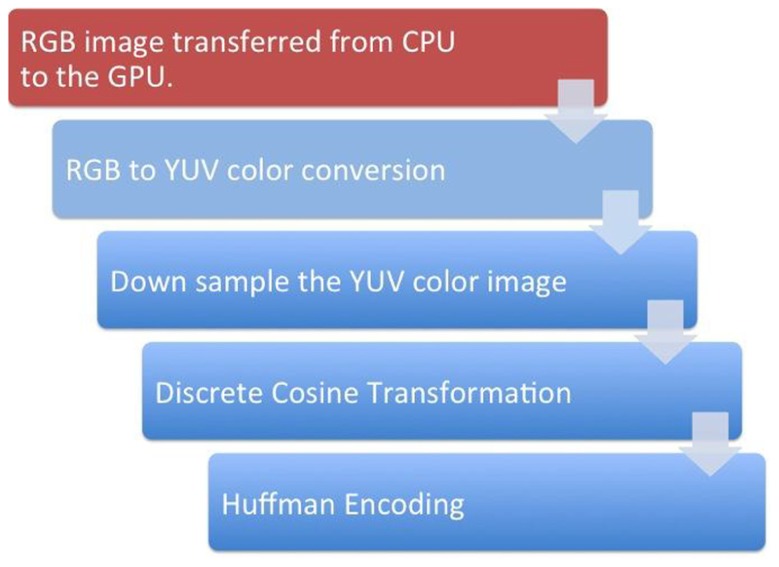
**A schematic representation of the steps involved in the GPU-based H.264 encoding**. The red box represents the steps that happen in the GPU while the blue boxes represent the steps that occur in the GPU.

The computational complexity of H.264 encoding would take a few seconds per frame if conducted using traditional compute processing units (CPU)-based architecture and so would degrade the real-time nature of the remote visualization (Wu et al., 2009). To alleviate this issue, we used a GPU-based H.264 video encoding process (Nvidia, 2012). **Figure 4** presents a schematic representation of the proposed encoding system. The first step converted a given 2D image from its native red green blue (RGB) format to the YUV image format. To achieve this with GPU acceleration, the given image was tiled at 8 × 8 pixels and the subsequent 64 RGB pixel values were loaded into shared memory in 64 parallel threads. The RGB values were stored in a 1D array in shared memory to allow simultaneous computations on the same data structure. The color conversion from RGB to YUV was implemented through three equations: *Y* = 0.29900 * *R* + 0.58700 * *G *+ 0.11400 * *B* - 128; *U* = -0.16874 * *R* - 0.33126 * *G* + 0.50000 * *B*; *V* = 0.50000 * *R* + 0.41869 * *G* - 0.08131 * *B*. The three equations were sequentially computed by one thread for each pixel. The next step was to downsample the YUV image. The downsampling of the *U* and *V* components was done by 32 threads in parallel. 16 threads computed the mean of a 2 × 2 pixel block for the *U* and the other 16 threads on the same pixel block for the *V* values. The mean was computed by adding all four values and dividing the result by 4. The division was replaced by a shifting operation to optimize performance. The next step was to store the down sampled YUV values in global memory. The *Y* values were stored in consecutive 8 × 8 pixel blocks, which were mapped to a 1D array. The same held for the *U* and the *V* components. However, the resulting down sampled 4 × 4 blocks were grouped to 8 × 8 blocks to prepare the data for a discrete cosine transformation (DCT).

For the DCT kernel, each component (*Y*, *U*, and *V*) was processed separately by one call to kernel 2. It had eight threads per block and did the following: loading the data was straightforward since it was already stored in the required 8 × 8 pixel blocks. Each of the eight threads loaded one row of a block into shared memory. To avoid the complex and sequential computation of a 2D DCT, the computation was split into several 1D separable DCTs. The computation of the DCT for each row was independent and was conducted by eight parallel threads. By using this optimized 1D DCT algorithm, the 2D DCT of an 8 × 8 pixel block was computed using 464 additions and 80 multiplications. Quantization was a simple division by the user-selected image compression quality factor for every coefficient in the 8 × 8 block. Quantization tables were similar for all blocks of a component. Before they were applied in the DCT kernel, the tables were multiplied by the scaling values of the DCT step as described before. The scaled and adapted quantization tables were applied to the coefficients by eight threads in parallel. Each thread computed one line of eight DCT coefficients and multiplies them by the inverse of the corresponding value in the quantization table.

The last step of the H.264 compression was Huffman encoding, which made use of the many zeros in lower frequencies resulting from DCT and quantization (Cheng et al., 2004). It included an entropy encoder that was used to encode the discrete wavelet transformation (DWT) coefficients plane-wise and an arithmetic encoding that was used for removing redundancy. Finally, a rate control algorithm was employed to truncate the data while keeping the image distortion low.

#### Jitter avoidance

Visualization using data streams is limited by frame-to-frame jitters that occur because of non-uniform network data transfer rates (Hamza-lup et al., 2007). Such jitters affect the real-time visualization perception for the client. Playout buffers are commonly used for alleviating jitter, where the video stream is buffered for a fixed amount of time before the client visualizes the frame (Li et al., 2008). The buffering time used for the playout buffer was adaptively varied in order to suit the client–server network connection. At each frame received by the client, the network delay *t* for a set of *N* frames was calculated by the client. A clustered temporal averaging that eliminated random fluctuations in the network delay was used to find the average network delay *t*^′^. The client then buffered the frames for the *t*^′^ time frame. The value of *N* was adaptively varied using a Go-Back *N* algorithm (Stevens, 2003) to account for temporal fluctuations in the network delay.

## RESULTS

**FIGURE 5:**
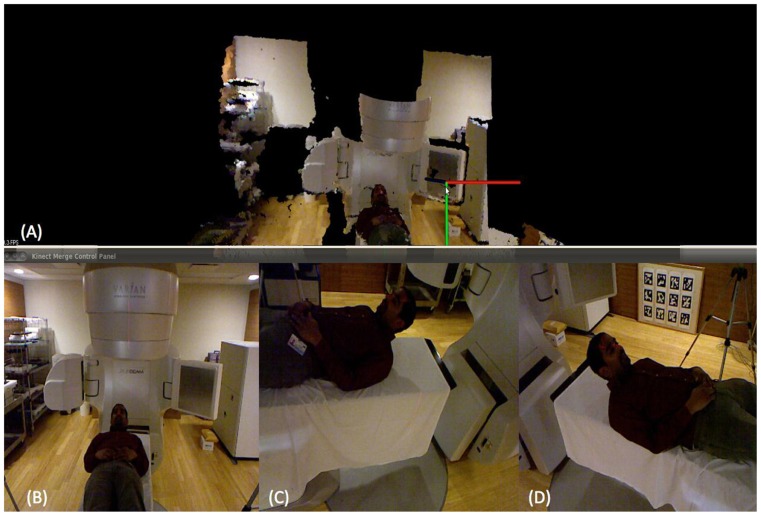
**3D head and neck surface acquisition at the server side in a normally lit environment**. The 3D rendering at the client side is shown in **(A)** while the three 3D cameras at the server side are shown in **(B)**, **(C)**, and **(D)**.

**FIGURE 6:**
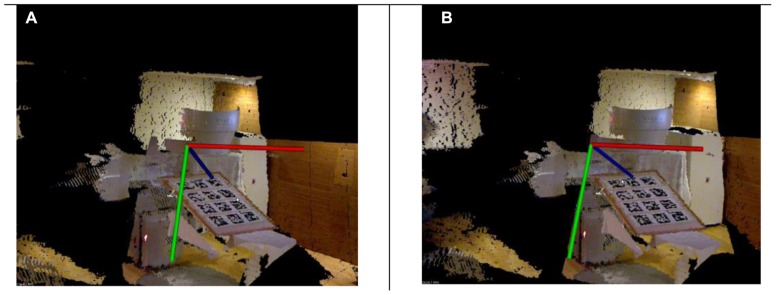
**3D stereo images generated for each of the eyes for a known view angle**. The subtle changes between the left and right eye images are shown with the coordinate system. Each of these images were encoded with H.264 standard at the server side and streamed to the client side for real-time visualization.

**Figure 5** illustrates a linear accelerator environment imaged using the proposed system. The 3D RGB images associated with each of the Kinect cameras used for this illustration are shown in the bottom of the image. Specifically, **Figures 5B**–**D** shows the subject’s position in the treatment couch as seen by the three Kinect cameras. The 3D cloud of surface voxels obtained from the co-registered camera system and visualized in 3D at the client side as shown in **Figure 5A**. The 3D view generated using each of the 3D camera’s information was then used to generate the two stereo pair images for each eye of the client. **Figures 6A,B** depict examples of the 2D stereo pairs during imaging of the camera calibration marker board. Such 3D content representing the patient and the treatment setup (**Figure 5A**) will be converted to 2D stereo pairs and will be visualized in 3D at the client side.

**Table 1** presents the hardware configuration used for the proposed client–server-based remote visualization framework. **Table 2** presents the results obtained for the remote visualization of the 3D treatment room space. Two image sizes, 1280 × 960 pixels and 640 × 480 pixels, were investigated for accessing the number of frame that can be transferred over a gigabit Ethernet connection. The usage of gigabit connection is to demonstrate a remote visualization within a given medical facility. The GPU-based H.264 was able to encode the large and small frames at rates of 14 and 4 ms, leading to transfer rates of 81 and 320 FPS per eye, respectively. Thus with a gigabit Ethernet connectivity, the client–server system was able to support real-time 3D treatment space visualization. **Table 3** presents the results obtained for the remote visualization using a 100 Mbps network with an effective streaming bandwidth of approximately 8 Mbps, simulating collaboration between two remotely located facilities. It can be seen that, given real-time constraints, a 640 × 480 stereo image size would provide 40 FPS, adequate for real-time visualization. Constraining the image size to be greater than 640 × 480 introduced jitter artifacts.

**Table 1:** System configuration.

3D Camera	Microsoft Kinect (6 cameras)
Server	Intel Core i7 3.6 Ghz, 8 GB RAM
Server GPU	Nvidia GTX 680m (2)
Network interface	Ethernet
Client	Intel Core i7 3.6 GHz, 8 GB RAM
3D display	Viewsonic 120 Hz LED display
3D wearable accessory	Nvidia 3D vision

**Table 2:** Remote visualization characteristics using a gigabit Ethernet connection.

RGB image size	1280 × 960 pixels	640 × 480 pixels
Stereo H.264 frame size	110 KB	28.5 KB
Stereo H.264 encoding time	14 ms	4 ms
Stereo image generation time	30 ms	30 ms
Effective streaming bandwidth	72 Mbps	72 Mbps
Frames transferred over network	~81 FPS	~320 FPS

**Table 3:** Remote visualization characteristics using a 100 Mbps connection with a frame rate of 30 FPS.

RGB image size	1280 × 960 pixels	640 × 480 pixels
Stereo H.264 frame size	110 KB	28.5 KB
Stereo H.264 encoding time	14 ms	4 ms
Effective streaming bandwidth	8 Mbps	8 Mbps
Frames transferred over network	~8 FPS	~40 FPS

## DISCUSSION

A framework for remote 3D visualization is presented in this paper. A multi-3D camera framework is used for acquiring the D treatment space. A client–server framework enables the 3D treatment space to be visualized by remotely located experts in real-time. The visualization tasks on the client side are offloaded into the server side to enable flexibility on the client side. A scalable multi GPU system that enables rendering the 3D treatment space in stereo and in real-time is employed on the server side. The rendered 3D images are then encoded using a GPU-based H.264 encoding for streaming purposes. Results showed that experts within a clinical facility and with high-speed gigabit Ethernet connectivity will be able to visualize the treatment space with 1280 × 960 pixel resolution at approximately 81 frames per second. For experts remotely located, the treatment space visualization can be conducted at 40 FPS with a resolution of 640 × 480 pixels.

Two technical limitations were observed in our client–server setup. The network bandwidth did not form a bottleneck for experts located in the same high-speed network and visualizing with a frame size of up to 1920 × 1080 pixels. The 3D treatment space acquisition, which occurred at a rate of 30 FPS formed the bottleneck in this case. The other key tasks such as 3D stereo rendering and H.264 encoding occurred at a rate faster than the treatment space acquisition rate. However, it was observed that for frame sizes greater than 2550 × 1940 pixels, the H.264 encoding took more time that the camera acquisition. Thus for greater frame sizes, offloading client tasks to the server led to an overall reduction in the speedup. Future work will focus on improving the H.264 encoding algorithm efficiency for stereoscopic video sequences.

The second limitation was that the number of server supported clients depended on the number of GPUs available for providing the required fast 3D rendering and encoding because each GPU was dedicated to handle a set of client tasks based on the requested frame size. Future work will focus on using a GPU cluster coupled using load-balancing algorithms that enable efficient GPU usage.

Jitter in the 3D visualization occurred for clients that used available bit-rate network connections. The jitter avoidance mechanism discussed in this paper removed such artifacts, but its effectiveness was limited by the network behavior. Increases in the network delays and packet loss rates led to random decrease in the *N* value and hampered the visualization system. Future work will focus on addressing such network connectivity issues.

## Conflict of Interest Statement

The authors declare that the research was conducted in the absence of any commercial or financial relationships that could be construed as a potential conflict of interest.
